# A Secure Secret Key Agreement Scheme among Multiple Twinning Superlattice PUF Holders

**DOI:** 10.3390/s23104704

**Published:** 2023-05-12

**Authors:** Jing Liu, Jianguo Xie, Junwei Zhang, Biao Liu, Xiaoming Chen, Huamin Feng

**Affiliations:** 1 School of Electronic Engineering, Beijing University of Posts and Telecommunications, Beijing 100876, China; liudingjing@bupt.edu.cn; 2 Beijing Electronic Science and Technology Institute, Beijing 100070, China; 3 State Key Laboratory of Integrated Service Networks and the School of Cyber Engineering, Xidian University, Xi’an 710126, China

**Keywords:** group key agreement, multiple twinning superlattice PUF, reusable fuzzy extractor

## Abstract

Modern cryptography attributes the security of a cryptographic system to the security of the key. How to securely distribute the key has always been a bottleneck in key management. This paper proposes a secure group key agreement scheme for multiple parties using a multiple twinning superlattice physical unclonable function (PUF) that can be synchronized. By sharing the challenge and helper data among multiple twinning superlattice PUF holders, the scheme employs a reusable fuzzy extractor to obtain the key locally. Moreover, adopting public-key encryption encrypts public data for establishing the subgroup key, which provides independent communication for the subgroup. At the same time, when the subgroup membership changes, the public key encrypts new public data to update the subgroup key, forming scalable group communication. This paper also presents a cost and formal security analysis, which shows that the proposed scheme can achieve computational security by applying the key obtained by the computationally secure reusable fuzzy extractor to the EAV-secure symmetric-key encryption, which has indistinguishable encryption in the presence of an eavesdropper. Additionally, the scheme is secure against physical attacks, man-in-the-middle attacks, and machine learning modeling attacks.

## 1. Introduction

Modern cryptography attributes the security of the cryptographic system to the security of the *key* using cryptographic algorithms and cryptographic protocols. Therefore, key management is an essential field of information security and a problematic issue in cryptography, and key generation and distribution are among the most relevant topics. The purpose of key management is to ensure the security of keys, that is, the authenticity and validity of keys. Key generation and distribution based on a physical unclonable function (PUF) are worldwide hotspot directions of information security technology to reduce key management risk and enhance security.

PUF is one typical representation of physical cryptography, with unique features such as ease to use, low cost, and power consumption [1,2]. During the chip-making, the process parameter for deviation or a deliberately introduced random factor causes a unique physical one-way function between the challenge (input) and the response (output) [3]. In addition, PUF is unique and unclonable, and even though under the same design scheme and manufacturing process, it is physically and mathematically unclonable. Because of these characteristics, PUF can be used as the key management device for key distribution, which is one of the mature applications of PUF [4].

Semiconductor superlattice (SSL) is a new PUF technology and a significant breakthrough in semiconductor physics and material science, and its development history is long and winding. SSL was proposed by Esaki and Tsu of IBM Lab in 1970 [5]. They theoretically anticipated that the differential conductance effect and cascade resonance tunneling effect are expected to realize high-frequency self-oscillation. Although there was an upsurge in SSL research at that time, it soon fell into a trough due to the consistency of the mass production of SSL devices. In 1996, Zhang et al. [6] first observed spontaneous chaos oscillation of electric current in SSL at low temperature (4.2 K). Since the chaos oscillation in SSL can only be realized at low temperatures, its application research has been stagnant for a long time. In 2012, Huang et al. achieved chaos oscillation at room temperature for the first time by improving the structure of superlattice materials and designed a physical random number generator with a speed of up to 80 Gbps [7,8].

**Figure 1:**
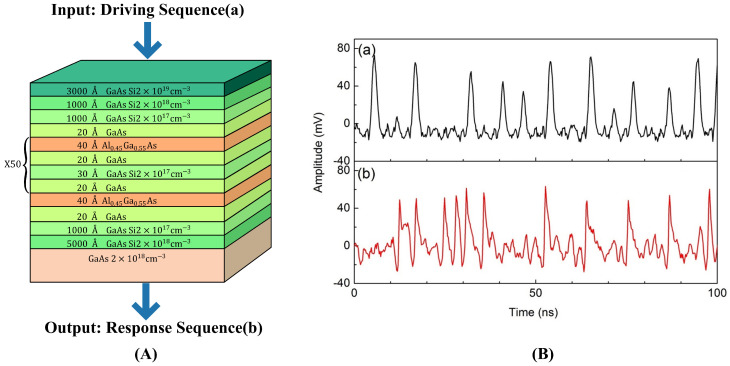
The structure (**A**) and the correlation between input and output (**B**) of GaAs/Al${}_{0.45}$Ga${}_{0.55}$As SSL.

After the developing spontaneous, chaos oscillation characteristics of SSL at room temperature, Li et al. successively discovered other physical phenomena [9,10,11,12,13], such as chaos synchronization and physical one-way function characteristics, etc. In particular, under DC bias, the input corresponds uniquely and stably to the output, a complex high-order nonlinear function of the input. Chen et al. [14] proposed the concept of superlattice PUF for the first time at the Xiangshan Science Conference in 2018. They elaborate that the PUF properties of SSL originated from 1. **Unclonability**. It is caused by the uncontrollable rise and fall of single atomic levels during the preparation of SSL growth, which is mainly reflected in the unclonability of wafers. Even with the same molecular layer-by-layer growth processes, it is impossible to produce identical wafers due to the unique growth dynamics and effects of the interface grading in the GaAs/(Al, Ga)As structure. 2. **Physical one-way function**. A noise injection can stimulate the chaos oscillations of SSL devices. Concerning challenge-response functionality, a motivation of certain continuous challenges can produce a corresponding continuous chaotic response (with a slight deviation). However, the correlation between the challenge signal and the response signal is very weak, as shown in Figure 1.

After the developing of the spontaneous, chaos oscillation characteristics of SSL at room temperature, Li et al. successively discovered other physical phenomena [9,10,11,12,13], such as chaos synchronization, physical one-way function characteristics, etc. In particular, under DC bias, the input corresponds uniquely and stably to the output, a complex high-order nonlinear function of the input. Chen et al. [14] proposed the concept of superlattice PUF for the first time at the Xiangshan Science Conference in 2018. They elaborated that the PUF properties of SSL originated from the following: 1. Unclonability. It is caused by the uncontrollable rising and falling of single atomic levels during the preparation of SSL growth, which is mainly reflected in the unclonability of wafers. Even with the same molecular layer-by-layer growth processes, it is impossible to produce identical wafers due to the unique growth dynamics and effects of interface grading in the GaAs/(Al, Ga)As structure. 2. Physical one-way function. A noise injection can stimulate the chaos oscillations of SSL devices. Concerning the challenge-response functionality, stimulation by certain continuous challenges can produce a corresponding continuous chaotic response (with a slight deviation). However, the correlation between the challenge signal and the response signal is very weak, as shown in Figure 1.

Superlattice PUFs have some other unique features. The superlattice physical functions of inter-wafer SSL devices vary, but the twinning ones of the same wafer have approximately identical physical functions. Twinning is a chaos synchronization phenomenon between SSL devices from the same wafer. According to this natural property of superlattice PUF, the idea of applying it to solve the key distribution problem has been proposed. In 2018, Liu et al. [15] proposed and experimentally proved a new point-to-point key distribution technology among two twinning superlattice PUFs with high throughput. On this basis, Wu et al. [16] experimentally demonstrated the long-distance public channel symmetric-key distribution scheme among two twinning superlattice PUFs between Suzhou and Beijing at a rate exceeding 7 Mbps.

However, with high concurrency and widespread access in interaction demands from users entering the Internet of Everything (IoE) world, security issues in the IoT environment have become increasingly severe, highlighting the advantages of secure group communication [17,18]. Group members use group keys for secure and lightweight communication, which meet security requirements and significantly reduce network overhead [19,20,21]. The point-to-point key agreement technique based on superlattice PUF shows that identical digital keys can be generated locally by both the sender and recipient, as each possesses a twinning superlattice PUF that can be synchronized. Compared to other key distribution methods, using the key agreement technique based on superlattice PUF provides a higher level of security since it ensures that no transfer of symmetric-key information occurs between the sender and recipient. Moreover, it is anticipated that utilizing the chaos synchronization phenomenon among multiple twinning superlattice PUFs can enable secure many-to-many key agreement technology.

This paper proposes a secure key agreement scheme based on chaos synchronization among multiple twinning superlattice PUFs driven by the same challenge the sender chooses to realize secure group communication. The group members are divided into the sender (key generator) and the recipient (key reconstructor). When one member becomes a sender, the remaining members take on the recipient role. Each group member holds a superlattice PUF from the same wafer that could be synchronized. Notably, the sender generates non-sensitive information data and sends it to the recipient. Using a reusable fuzzy extractor, the same digital key is generated locally by the sender and the recipient. The public channel only transmits the challenge and helper data, which do not disclose information about the secure keys and are impervious to interception and tampering by eavesdroppers. In order to ensure the security of the helper data after reuse, this paper uses the reusable fuzzy extractor to complete the key reconstruction. Additionally, the security analysis demonstrates that combining the computational security reusable fuzzy extractor and an EAV-secure private-key encryption scheme is also a computational security encryption scheme.

Furthermore, When the group member desires to establish subgroup communication, this paper adopts public-key encryption to encrypt the public challenge and helper data to prevent members from outside the subgroup. The other advantage is that only lightweight computations are necessary to complete the re-keying process, guaranteeing forward and backward secrecy while reducing communication and computing overhead.

The remainder of this article is structured as follows. Section 2 describes related work on existing group communication approaches. A preliminary, including secure group communication and fuzzy extractor, is presented in Section 3. Section 4 offers the scheme details for implementing the lightweight key agreement. Section 5 presents the security proof of the proposed scheme. Section 6 discusses limitations and future work. And Section 7 presents the conclusion part of this study.

## 2. Related Work

Up to now, many schemes have been proposed for key distribution/agreement in secure group communication. This section enumerates several typical approaches based on whether PUF is used.

Liu et al. [22] proposed a group key distribution protocol with unconditional security, which utilizes the Chinese remainder theorem (CRT). In the registration stage, the key distribution center (KDC) establishes a private shared secret with each member. Members can recover the group key by combining the private shared secret with the KDC and the public share obtained through broadcasting.

Ref.  [23] proposed a scalable key management scheme based on distributed trees. The scheme trusts all senders equally and supports many-to-many communication. The paper adopts the Logical Key Hierarchy (LKH) to complete the group key agreement by mixing the blind keys of the two sibling nodes with one-way functions. However, the description of the transmission channel of the blind key and the selection of the one-way function is insufficient.

Mahalle et al. [24] used Paillier threshold cryptography based on Shamir’s secret to distribute the group key. In this method, the server generates a public key for itself and multiple private keys for nodes which are distributed to the nodes securely. The server generates a session secret and a random value as an encryption key for the session secret when a member initiates a group activity. The session secret is then shared among all members. However, all members need to be authenticated before sharing. After successful verification, the server encrypts the key with the public key, which provides the required security as the complete private key can only decrypt it.

To prevent illegal members from obtaining the group key, Ref. [25] proposed a centralized approach where the server distributes a temporary group key to members. The group key is generated by merging the secrets of group members. Upon receiving the temporary group key from the server, the nodes perform a series of operations to derive the actual group key. The nodes store the temporary group key and compute the actual group key from it before encrypting communication messages. This approach ensures that the actual group key is not stored on the nodes, reducing the risk of key exposure.

Dong et al. [26] used a strong PUF as a control unit and, together with sensors, formed a body area network (BAN) for group communication. The control unit cu and each sensor $s_{i}$ hold the different PUF. When a group of sensors is formed, the control unit generates a group key using the PUF $F_{cu}\left(\right)$ with the group name $G_{N}$ as input and distributes it to each sensor after encrypting it. Each sensor inputs the group name into its PUF $F_{s_{i}}$ to produce an output that differs from the group key and then stores it after performing an XOR with the actual group key to increase security. Use the sensor’s PUF to instantly generate an output when it wants to communicate with the others, then XOR the result to find the group key.

Huang et al. [27] proposed a group key distribution scheme with mutual authentication for wireless sensor networks (WSNs), which combines software-defined networking (SDN) and PUFs. This scheme constructs a group key distribution model, including the control and data planes. The control plane includes the Main Controller (MC) and Auxiliary Controller (AC), and the data plane comprises various sensor nodes. The MC stores the initial challenge-response pairs (CRP) of all sensor nodes in the security database and generates key factors with the responses of each node. Then MC chooses an optimal path for distributing the group key, finding an AC closest to each node and distributing the key factors to it. After the node runs its PUF module and gets the response, it XORs with the key factor to generate the key in real-time.

From state-of-the-art, nodes mainly store the temporary group key to ensure security in the group key distribution/agreement scheme. Then they recover the true group key when using it through some simple operations. The key distribution/agreement system adds a layer of security using PUF. To get the info necessary to recover the group key, an attacker needs access to the PUF module and storage. The current approaches still have a flaw. In most cases, the key distribution or agreement scheme employs the preset or public key encrypted transmission technique, inconveniences key changes, and increases the likelihood of key disclosure. According to [15], there is no need to preset keys using chaos synchronization among twinning superlattice PUFs for key distribution/agreement. Meanwhile, the security of the key agreement is ensured as the symmetric digital key is not transferred between the nodes.

## 3. Preliminary and Background

### 3.1. Secure Communication Group

Group communication provides efficient communication between multiple devices, saving the cost of reaching a shared key for point-to-point communication. During group key generation, it is necessary to ensure that each member can receive or calculate the group key in a secure and scalable manner. Furthermore, users not in the communication group cannot obtain the group key. There are three main architectures for group communication [28]:

Group communication provides efficient communication between multiple devices, saving the cost of reaching a shared key for point-to-point communication. During group key generation, it is necessary to ensure that each member can receive or calculate the group key securely and efficiently. Furthermore, users not in the communication group cannot obtain the group key. There are three main architectures for group communication [28]:

Centralized: A single entity controls the entire communication group and distributes the group key or the material needed to form the group key.

Decentralized: A group is divided into several subgroups. Each subgroup leader generates required materials about the group key and distributes them to nodes. Each team leader is given a share of the load, reducing the pressure on the server.

Distributed: Each member has the same responsibilities and status. There is no group leader in the distributed architecture. Each member shares information with other members to agree on the group key. Compared with centralized and decentralized architecture, distributed architecture needs more network messages and computations.

The point to remember is that the communication group may be dynamic. The group key needs to be updated to ensure that any user outside the group does not obtain the messages transmitted between the communication group, called re-keying [29]. Among them, the dynamic changes of the group include the joining of new members or the leaving of current members. When a new member joins the group at time *t*, the group updates the key to ensure that the new member cannot obtain the communication message before *t*, called forward secrecy. In contrast, when the current member leaves at time *t*, the group updates the group key to ensure that the leaving member no longer has access to communication messages after *t*, known as backward secrecy.

### 3.2. Fuzzy Extractor

A fuzzy extractor is a cryptographic method for extracting a uniformly random string and accurately recoverable from a noisy random source. It primarily executes two functions: information reconciliation, which turns similar information into the same information, and privacy amplification, which turns ununiformly distributed strings into uniformly distribute strings. In 2004, Dodis et al. [30] proposed the fuzzy extractor, which can be applied to cryptosystems such as key agreement, symmetric key generation, and public key.

**Figure 2:**
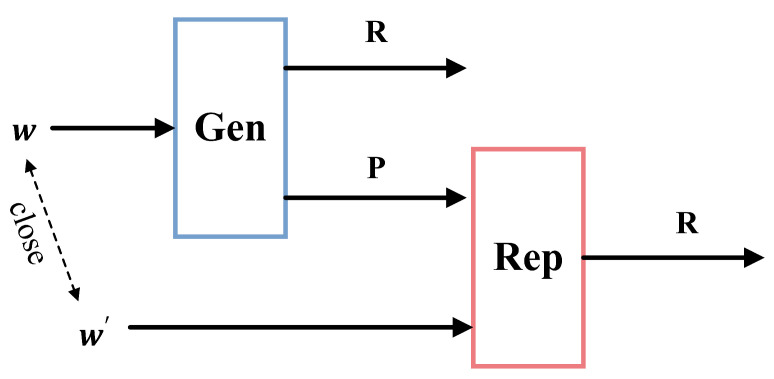
The structure of fuzzy extractor.

Fuzzy extractors similarly consist of a pair of procedures, as shown in Figure 2:

(1) In the generation procedure (Gen): a uniformly random string *R* and a public helper value *P* are produced from a source value *w*.
(2) In the reproduction procedure (Rep): the original string *R* is reproduced by using the helper value *P* and a close value $w^{\prime }$.

Correct reproduction of *R* by Rep is guaranteed as long as the source value $w^{{}^{\prime }}$ is within a certain distance *t* from the source value *w*, where distance can be measured by some metric such as Hamming distance [30,31].

### 3.3. ElGamal Encryption

ElGamal proposed a public-key cryptosystem that relies on the difficulty of solving the Discrete Logarithm problem in the multiplicative group modulo a prime *p* denoted as $\left(Z_{p}^{*},\cdot \right)$. To ensure the security of the ElGamal Cryptosystem, it is essential that the Discrete Logarithm problem in $Z_{p}^{*}$ is infeasible. In the ElGamal Algorithm 1, the plaintext *x* is multiplied by a random value $\beta^{k}$ to produce the masked value $y_{2}$, and $\alpha^{k}$ is transmitted as part of the ciphertext. Bob, who knows the private key *a*, can calculate $\beta^{k}$ from $\alpha^{k}$ and then divide $y_{2}$ by $\beta^{k}$ to remove the mask and recover the original plaintext *x*.

**Algorithm 1** ElGamal Public-key Cryptosystem in multiplicative group $Z_{p}^{*}$Assuming that the Discrete Logarithm problem in the multiplicative group $\left(Z_{p}^{*},\cdot \right)$ is infeasible, ElGamal Cryptosystem can be used to encrypt and decrypt messages. The public key is comprised of three values: a prime number *p*, a primitive root α in the multiplicative group $\left(Z_{p}^{*},\cdot \right)$, and $$\beta \equiv \alpha^{a}\;\left(\mathrm{mod}\;p\right),$$
where *a* is the private key. To encrypt a message *x*, a random number *k* is chosen by Alice, and the resulting ciphertext is $\left(y_{1},y_{2}\right)$, where $$y_{1}\equiv \alpha^{k}\;\left(\mathrm{mod}\;p\right)$$
and $$y_{2}\equiv x\beta^{k}\;\left(\mathrm{mod}\;p\right).$$
To decrypt the ciphertext, Bob uses the private key *a* to compute $\beta^{k}\equiv \left(y_{1}^{a}\right)\;\left(\mathrm{mod}\;p\right)$
and then computes the plaintext message as $x\equiv y_{2}{\left(y_{1}^{a}\right)}^{-1}\;\left(\mathrm{mod}\;p\right).$

## 4. Key Agreement Scheme Based on Multiple Twinning Superlattice PUFs

### 4.1. System and Threat Model

A secure key agreement is achieved in the group communication system through the combination of upper computer software and a multi-twinning superlattice PUF hardware module possessed by each legal user. That allows for the exchange of message between legal users. When the group communication members join or leave the subgroup, the proposed scheme changes the subgroup key. Therefore, the attacker *Adver* has the following attack ability in the above group communication system:

(1) Impersonation attack. Assuming that Alice and Bob are legal communication parties, Alice wants to establish a session key with Bob. However, she is concerned that she may communicate with an attacker *Adver* impersonating Bob.
(2) Replay attack. While agreeing on a group key between Alice and multiple legal communication parties, *Adver* may intercept the incentives sent between them and use the last challenge sequence to replay, trying to communicate them with the old group key.
(3) Man-in-the-middle attack. *Adver* may intercept the message sent by Alice and tamper with it. It is then broadcast to the receiver to establish a new group key between him and other recipients.
(4) *Adver* changes the information sent by Alice on the public channel. Adver may modify the challenge sequence, which causes incentive errors, thus rejecting the key agreement. *Adver* may also modify the helper data. Suppose the modified number of bits causes the number of codeword errors to be less than the error correction ability *t*. In that case, the honest communication party can still obtain an unconditionally secure key. Suppose the modified number of bits causes the number of codeword errors to be greater than the error correction capability *t*. In that case, the honest communication party refuses the key agreement service.

In summary, this paper aims to propose a multi-party key agreement scheme based on multi-twinning superlattice PUFs on the same wafer, which can resist impersonation attacks, replay attacks, and man-in-the-middle attacks.

### 4.2. Key Agreemnet Scheme

**Figure 3:**
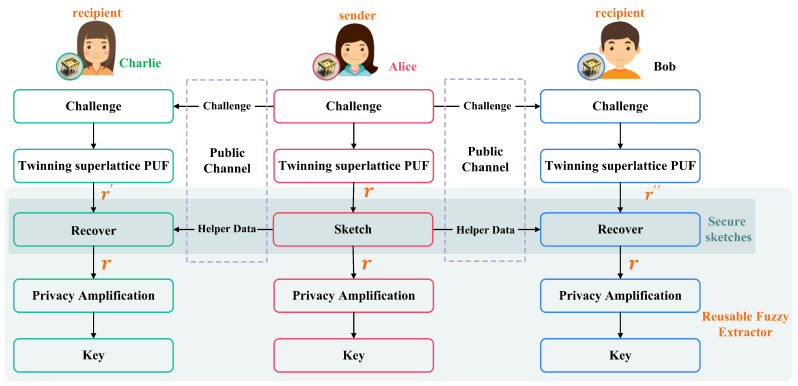
The key agreement protocol based on multiple twinning superlattice PUFs holders.

This paper proposes and demonstrates a multi-party key agreement scheme for multiple twinning superlattice PUFs holders from the same wafer driven by a synchronization challenge, as shown in Figure 3. Assume that multiple twinning superlattice PUFs are distributed to legal users and form a communication group. When establishing the group key, they are divided into two roles: the sender (key generator), usually only one, and the recipient (key reconstructor), the remaining group members, except for the sender. In Figure 3, Alice is the sender, the key generator, and Bob and Charlie are the recipients, the key reconstructor. Alice, Bob, and Charlie generate the key locally due to the twinning superlattice PUF they hold. Furthermore, any information about the key would not be disclosed, ensuring the security of the key agreement.

The following steps are required for group key agreement:

Step 1:Alice randomly chooses a challenge and obtains the response *w* through superlattice PUF model. *w* also is the input of the reusable fuzzy extractor. Subsequently, the generating algorithm *Gen* of the fuzzy extractor outputs a uniformly random string *R* and a public string *P* as the helper data.

Step 2: Alice sends the challenge and the public string *P* to Bob and Charlie. Then, Bob and Charlie input the challenge sequence into its superlattice PUF model and get the response $w_{i}$ slightly different from *w*. With the help of the helper data *P*, Bob and Charlie obtain the uniformly random string *R*, equal to Alice, through the reproduction algorithm *Rep* of the reusable fuzzy extractor.

Step 3: According to the entropy loss leaked by the public string *P* and the min-entropy of superlattice PUF, Alice, Bob, and Charlie get a short key *K* through *privacy amplification*.

Step 4: Repeating steps 1–3, a high-speed physical key stream can continuously be generated.

### 4.3. The Choice of Fuzzy Extractor

Due to inter-device variances and random external noises, the twinning superlattice PUF generates slightly different outputs even input the same challenge. The fuzzy extractor is used between the sender and the recipients to produce identical output from the twinning superlattice PUFs. Properly designed fuzzy extractors would not compromise the security of key distribution [32,33,34]. However, when applying the actual application scenarios, there are still certain limitations: the fuzzy extractor can only guarantee the security of extracting the key from the noise source once instead of multiple times.

**Figure 4:**
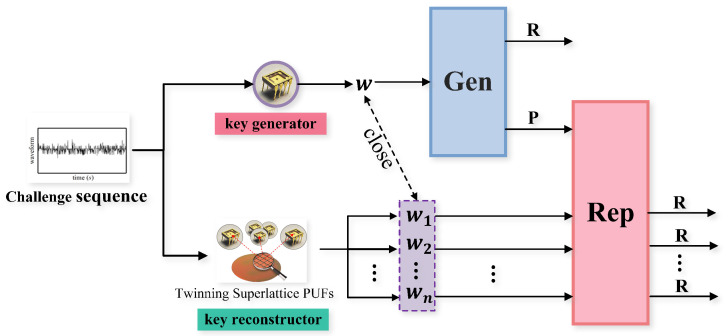
The schematic diagram of key agreement protocol based on multiple twinning superlattice PUFs.

Unlike point-to-point key distribution, this paper adopts the reusable fuzzy extractor to avoid security risks caused by multiple usages of challenge sequence and helper data. A fuzzy extractor is said to be reusable if it can be used to generate the same secure key from multiple registrations of the same biometric or PUF without sacrificing the security of the system [35,36,37]. In other words, a reusable fuzzy extractor allows users to use the same biometric or PUF for different applications or services while ensuring that each application or service has a unique and secure key. Figure 4 shows the schematic diagram for employing the reusable fuzzy extractor to obtain key agreement between multiple twinning superlattice PUFs. The key generator and each key reconstructor hold the twinning superlattice PUF based on chaos synchronization. They input the same challenge as an incentive and get similar responses. The key generator uses the *Gen* procedure of the reusable fuzzy extractor to produce helper data *P* and the random uniform string *R*. The key reconstructor obtains the string *R* consistent with the key generator through *Rep* procedure with the helper data *P* and the similar response $w_{i}$.

The reusable fuzzy extractor used in this paper is the digital locker based on the Sample-then-Lock proposed by Canetti et al. [38]. They design the first reusable fuzzy extractor without presumptions regarding the correlation between different sources. The extractor provides computational security within the digital lockers and can handle binary strings with near-linear error rates with Hamming noise. It is guaranteed that *R* can be fully recovered, meaning the recipient will obtain a perfectly identical copy of *R*, on condition that the Hamming distance between *w* and $w_{i}$ is less than the maximum number of correctable errors.

The construction of the Sample-then-Lock is briefly introduced below.

Sample-then-Lock: The fuzzy extractor utilizes sources that provide high-entropy samples. Specifically, for certain parameters *k* and α, the source *W* is considered to have α-entropy with *k*-samples if for randomly selected indices 1≤j1,⋯,jk≤n, the conditional min-entropy of *W* given $j_{1},\cdots ,j_{k}$ is at least α. Suppose that the fuzzy extractor output the random value *r*. To hide *r*, the Sample-then-Lock constructs a digital locker through $v_{1}$, samples a subset from symbols at random $v_{1}=w_{j1},\cdots ,w_{jk}$. Repeat the random sampling process until it results in a certain number *l* of digital lockers containing *r* and can be unlocked using $v_{1},\cdots ,v_{l}$ as keys. Using composable digital lockers makes it possible to sample more than once because only the individual entropy of $v_{i}$ needs to be argued. Reusability is made possible via composability.

The formal definition of the reusable fuzzy extractor is: Consider an alphabet Z and a source $W=W_{1},\cdots ,W_{n}$ with α-entropy *k*-samples, where each $W_{j}$ is a symbol from Z. Let (lock, unlock) be a digital locker scheme that is *l*-composable and has an error tolerance of γ, where keys and values are *k*-bit strings from $Z^{k}$. Define Gen, Rep as:

**Gen**

1. *Input: $w=w_{1},\cdots ,w_{n}$*
2. *Sample: $r\overset{\$}{\leftarrow }{\left\{0,1\right\}}^{k}$*
3. *For i=1,⋯,l:.*
  (i) *Choose random $1\leq j_{i,1},\cdots ,j_{i,k}\leq n$.*
  (ii) *Set $v_{i}=w_{j_{i,1}},\cdots ,w_{j_{i,k}}$*.
  (iii) *Set $c_{i}=lock\left(v_{i},r\right)$.*
4. *Output (r,p), where $p=p_{1}\cdots p_{l}$.*

**Rep**

1. *Input: ($w^{{}^{\prime }}=w_{1}^{{}^{\prime }},\cdots ,w_{n}^{{}^{\prime }},p=p_{1}\cdots p_{l}$)*
2. *For i=1,⋯,l:*
  (i) *Parse $p_{i}=c_{i}\left(j_{i,1},\cdots ,j_{i,k}\right).$*
  (ii) *Set $v_{i}^{{}^{\prime }}=w_{j_{i,1}}^{{}^{\prime }},\cdots ,w_{j_{i,k}}^{{}^{\prime }}$.*
  (iii) *Set $r_{i}=$* **unlock** $\left(v_{i}^{{}^{\prime }},c_{i}\right)$. *If $r_{i}\neq ⊥$ output $r_{i}$.*
3. *Output* ⊥.

### 4.4. Subgroup Communication

**Figure 5:**
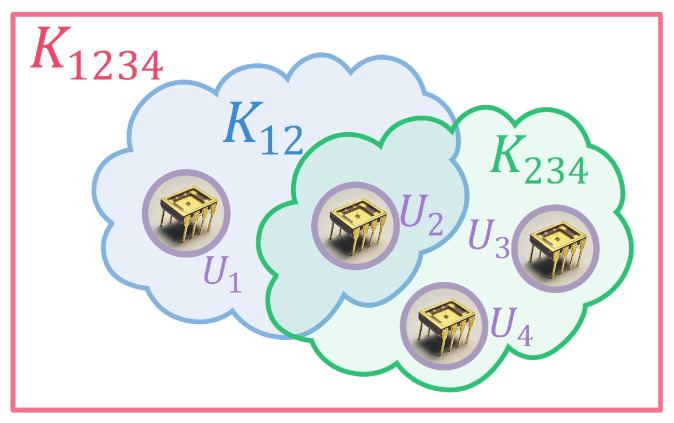
The key graph of the secure communication group.

Making a subgroup key for group members who wish to communicate privately is required to prevent other members from listening to challenge and helper data. As shown in Figure 5, there has the subgroup key and group key in the secure communication group, forming a key graph [39]. Let $U_{i}$ represent each member node, $U_{ij}$ represent the subgroup composed of $U_{i}$ and $U_{j}$, and $K_{ij}$ represents the subgroup key of user *i* and user *j*. In detail, member nodes include $U_{1}$, $U_{2}$, $U_{3}$, and $U_{4}$ in the secure communication group $U_{1234}$, and they share the group key $K_{1234}$. $U_{1}$ and $U_{2}$ form a subgroup $U_{12}$ and use the key $K_{12}$ for subgroup communication among them. So are $U_{2}$, $U_{3}$, and $U_{4}$. Since the superlattice PUF held by each member of the communication group has a chaos synchronization phenomenon, the challenge and helper data need to be transmitted in secret when establishing the subgroup key.

**Figure 6:**
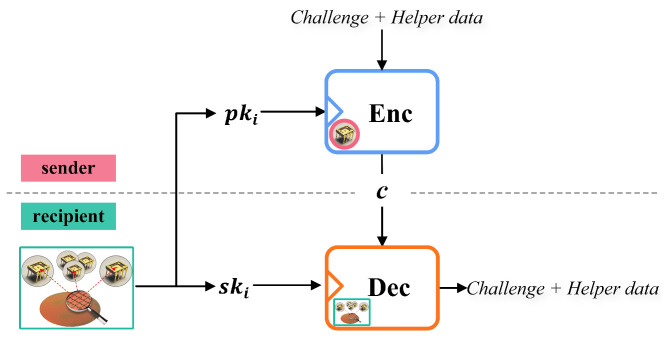
Encrypt the *Challenge* and *HelperData* with the public-key encryption scheme.

This paper uses the ElGamal encryption scheme based on the intractable discrete logarithm problem to protect public data. As shown in Figure 6, each recipient generates the public-private key pairs using the superlattice PUF as the random number generator.

### 4.5. Dynamic Group Management

The simplicity of group key update is another advantage of encrypting the challenge and helper data. Since communication groups are not always static, the group key must be updated to ensure forward and backward secrecy as group membership changes. Since the members holding twinning superlattice PUFs form a communication group, joining and leaving operations in dynamic group management are only available for subgroups.

#### 4.5.1. Member Join

**Figure 7:**
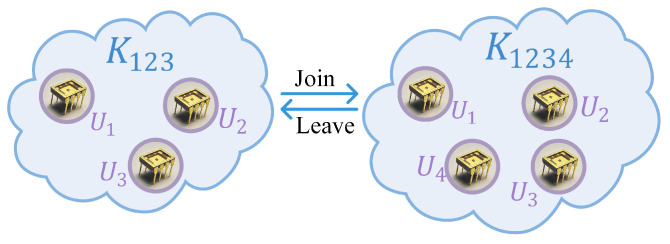
Star key graphs before and after a join (leave) request.

As shown in Figure 7, when $U_{4}$ joins the communication subgroup $U_{123}$ at time *t*, to ensure forward secrecy, the current group key must be updated: $K_{123}\to K_{1234}$, to prevent $U_{4}$ obtains the messages transmitted within the time $t^{{}^{\prime }}<t$.

The process for a new user to join any subgroup is as follows:

(1). A new user requests any member $U_{i}$ in the subgroup to join and generates its public-private key pair.
(2). $U_{i}$ randomly reselects the challenge *c*, generate the response *w* through the superlattice PUF. Then, through the reusable fuzzy extractor, $U_{i}$ generates the uniformly random string *R* and the public helper string *P*. $U_{i}$ encrypts *c* and *P* using the public key of the remaining members and sends the results to them, respectively.
(3). The remaining members decrypt the message with their private key to get *c* and *P*. Through the reusable fuzzy extractor, they can get *R* consistent with $U_{i}$.
(4). According to the entropy loss leaked by the public string *P* and the min-entropy of superlattice PUF, the members in the new subgroup get a short key *K* through privacy amplification.

For example, as shown in Figure 7, $U_{4}$ wants to join the subgroup $U_{123}$. Without loss of generality, suppose $U_{4}$ makes a join request to $U_{1}$. $U_{1}$ randomly reselects a challenge sequence $c^{{}^{\prime }}$ and gets *w* through superlattice PUF and $R^{{}^{\prime }}$ and $P^{{}^{\prime }}$ through reusable fuzzy extractor. Then $U_{1}$ uses the public key $PubK_{2}$ of $U_{2}$, $PubK_{3}$ of $U_{3}$ and $PubK_{4}$ of $U_{4}$ to encrypt $c^{{}^{\prime }}$ and $P^{{}^{\prime }}$ and sends the results to $U_{2}$, $U_{3}$, and $U_{4}$, respectively.

$$\begin{array}{cc} U_{1}\to U_{2} & :E_{PubK_{2}}(c^{{}^{\prime }}\left|\right|P^{{}^{\prime }})\\ U_{1}\to U_{3} & :E_{PubK_{3}}(c^{{}^{\prime }}\left|\right|P^{{}^{\prime }})\\ U_{1}\to U_{4} & :E_{PubK_{4}}(c^{{}^{\prime }}\left|\right|P^{{}^{\prime }}). \end{array}$$

$U_{2},U_{3}$, and $U_{4}$ use their private key to decrypt the message to get the challenge $c^{{}^{\prime }}$ and helper data $P^{{}^{\prime }}$.

$$\begin{array}{cc} U_{2} & :D_{PriK_{2}}(E_{PubK_{2}}(c^{{}^{\prime }}\left|\right|P^{{}^{\prime }}))\\ U_{3} & :D_{PriK_{3}}(E_{PubK_{3}}(c^{{}^{\prime }}\left|\right|P^{{}^{\prime }}))\\ U_{4} & :D_{PriK_{4}}(E_{PubK_{4}}(c^{{}^{\prime }}\left|\right|P^{{}^{\prime }})). \end{array}$$

Put $c^{{}^{\prime }}$ and $P^{{}^{\prime }}$ into their superlattice PUF to obtain the response $w_{i}$ that is slightly different from *w*. Then put $w_{i}$ and $P^{{}^{\prime }}$ into reusable fuzzy extractor, they get $R^{{}^{\prime }}$.

#### 4.5.2. Member Leave

When the $U_{i}$ leaves the subgroup at time *t*, to ensure backward secrecy, the current group key must be updated: $K_{123}\to K_{12}$, to prevent $U_{3}$ obtains the messages transmitted within the time $t^{{}^{\prime }}>t$. Like new member joining, public key encryption ensures that the new challenge $c^{{}^{\prime }}$ and helper data $P^{{}^{\prime }}$ can not be acquired by the leaving member, ensuring the leaving member will not acquire the session key. The process of group key update after members leave is similar to group initialization, which is equivalent to re-establishing a new secure communication group without the leaving member. Any group member assumes the sender, and the remaining members assume the recipient such that a new session is established, excluding the leaving members.

Similarly, $U_{4}$ wants to leave the right subgroup $U_{1234}$ as shown in Figure 7. The subgroup key must be updated: $K_{1234}\to K_{123}$. Without loss of generality, $U_{1}$ reselects a challenge $c^{{}^{\prime }}$ and gets the $R^{{}^{\prime }}$ and $P^{{}^{\prime }}$ through reusable fuzzy extractor. Then $U_{1}$ uses the public key $PubK_{2}$ and $PubK_{3}$ to encrypt $c^{{}^{\prime }}$ and $P^{{}^{\prime }}$ and sends the results to $U_{2}$ and $U_{3}$, respectively.

$$\begin{array}{cc} U_{1}\to U_{2} & :E_{PubK_{2}}(c^{{}^{\prime }}\left|\right|P^{{}^{\prime }})\\ U_{1}\to U_{3} & :E_{PubK_{3}}(c^{{}^{\prime }}\left|\right|P^{{}^{\prime }}). \end{array}$$

$U_{2}$ and $U_{3}$ use the private key to decrypt the message to get the challenge $c^{{}^{\prime }}$ and helper data $P^{{}^{\prime }}$.

$$\begin{array}{cc} U_{2} & :D_{PriK_{2}}(E_{PubK_{2}}(c^{{}^{\prime }}\left|\right|P^{{}^{\prime }}))\\ U_{3} & :D_{PriK_{3}}(E_{PubK_{3}}(c^{{}^{\prime }}\left|\right|P^{{}^{\prime }})). \end{array}$$

Put $c^{{}^{\prime }}$ and $P^{{}^{\prime }}$ into the superlattice PUF they hold to obtain the response $w_{i}$ that is slightly different from *w*. Then put $w_{i}$ and $P^{{}^{\prime }}$ into reusable fuzzy extractor, they get $R^{{}^{\prime }}$.

An approximation of the computing expenses of members is estimating the number of key encryptions and decryptions required by a join/leave request. The member who asks to join or leave is referred to as the requesting user, the member who started the subgroup key update is referred to as the initiator, and the other users in the subgroup are the nonrequesting users. Table 1 tabulates the cost of members for a join/leave request.

### 4.6. Computational Cost

Assume that there are *n* nodes in the communication group. Each node performs (*n*) PUF and (*n*) reusable fuzzy extractor operations while establishing the group key. The reusable fuzzy extractor operation includes an error correction using polar code $T_{Polar}$, an Error Correcting Code $T_{ECC}$, a general hash function $T_{UHF}$, and a digital lock operation $T_{DL}$. When establishing a subgroup key and changing subgroup members, two additional public key encryption and decryption operations are required compared to establishing a group key. We provide the execution times for establishing the group key and dynamic member management of the various protocols, as shown in Table 2. It is assumed that $T_{PUF}$, $T_{RFE}$, $T_{XOR}$, $T_{HMAC}$, $T_{E}$, $T_{D}$, $T_{Mod}$, $T_{Mix}$, and $T_{E/D}$ denote the computational cost required for the PUF operation, the reusable fuzzy extractor operation, an XOR operation, a hashed MAC operation, a symmetric encryption operation, a symmetric decryption operation, a modulo operation, a Mixing function operation, and the public-key cryptography using ElGamal cryptosystem, respectively. By the way, *m* represents the height of the key tree.

### 4.7. Experimental Results

**Figure 8:**
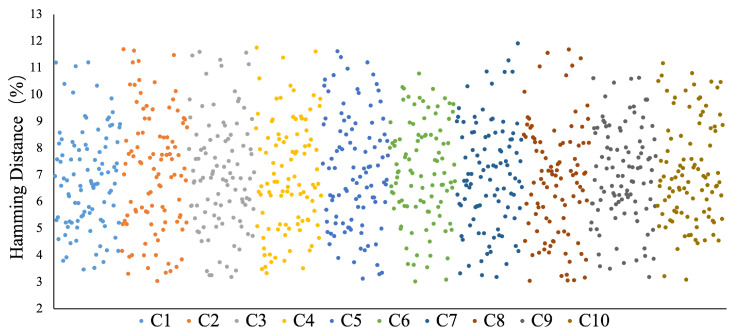
The Hamming distance of multiple twinning superlattice PUF pairs $(S_{1},S_{i}),i=2,\ldots ,100$. $C_{i},i=1,\ldots ,10$ represents the challenge used for each data set.

A superlattice is an analog device. Therefore, the input of the superlattice needs to be converted from a digital signal to an analog signal by a digital-to-analog converter (DAC) to excite the superlattice to generate an analog output signal. Subsequently, the analog output signal is converted into a digital signal by an analog-to-digital converter (ADC) and transmitted to the upper computer system. In order to realize the function of the reusable fuzzy extractor, this paper adopts an ARM-A9 embedded CPU as the host computer system in the experiment. We randomly select 100 multiple twinning superlattice PUFs, $S_{i},i=1,\cdots ,100$, for group communication simulation experiments. We use ten challenges, $C_{i},i=1,..,10$, with 64,800 bits as the input of the multiple twinning superlattice PUFs to obtain an output with 64,800 bits. Then, we evaluate the Hamming distance of the output, which is essential in determining the error correction code rate and the final secure key rate. Without loss of generality, we randomly choose a superlattice PUF and plot the Hamming distance between it and the remaining 99 superlattice PUFs in Figure 8. As shown in Figure 8, the ordinate represents the Hamming distance between every two twinning superlattice PUFs, the abscissa represents two PUF pairs, $(S_{1},S_{i}),i=2,\cdots ,100$, and ten colors represent the results of ten data sets. The Hamming distance is mainly distributed between 3% and 12%.

The low-density parity-check (LDPC) codes supported by the DVB-S2 standard [40] are subsequently incorporated as the error correction of the reusable fuzzy extractor. With a 1/4 coding rate and a codeword length of 64,800 bits, redundant error correction against burst errors of 13% is possible.

Since the experiment is conducted on a local area network, the communication time cost is negligible. In order to accurately test the final secure key rate, we set the key agreement quantities to 100 Mb. Through multiple experiments, we find that the average time it takes for the key generator initiates a group key construction request to all members in the group to reach the same key (100 Mb) is 16.8 s. Therefore, the final secure key rate is 100 Mb/16.8 s ≈ 5.95 Mbps.

## 5. Security Analysis

### 5.1. Theoretical Security of the Scheme

The computational security of Sample-then-Lock: Canetti et al. [38] show that the reusability of Sample-then-Lock follows from the composability of digital clockers. Moreover, it is computationally secure under tolerating near-linear error rates. Tolerating the near-linear error rate implies that Sample-then-Lock has *t* number of error corrections, where t/n=O(c) and *c* is a constant, conditional on input length *n*. Computational security means that breaking it using the current best methods requires computation far beyond the attacker’s computational resources. Meanwhile, Canetti et al. prove that an unbounded time simulator *S* cannot distinguish between *R* and *U*, *U* is an independent uniform random variable over ${\left\{0,1\right\}}^{\kappa }$ as shown in Proposition 1. In other words, the simulator *S* has a negligible probability of guessing the key under limited computing power.

**Proposition** **1** ([38]). *Let U denote the uniform distribution over ${\left\{0,1\right\}}^{\kappa }$. Then*

$$\begin{array}{c} \left|E[S^{{\left\{idealUnlock\left(v_{i},r\right)\right\}}_{i=1}^{l}}\left(R,{\left\{j_{i,1},\cdots ,j_{i,k}\right\}}_{i=1}^{l}\right)\right]\\ -E[S^{{\left\{idealUnlock\left(v_{i},r\right)\right\}}_{i=1}^{l}}\left(U,{\left\{j_{i,1},\cdots ,j_{i,k}\right\}}_{i=1}^{l}\right)]|\\ \leq \frac{q(q+1)}{2^{\alpha }}\leq \frac{1}{3p(\lambda )}, \end{array}$$

*where α is the entropy of source, and q is the maximum number of queries S can make.*

After the key is obtained by the protocol outlined in this paper, any *EAV*-secure private-key encryption schemes can be used to complete the secrecy communication. The remainder of this section will prove that combining the computational security reusable fuzzy extractor (Sample-then-Lock) and an *EAV*-secure private-key encryption scheme is also a computational secure encryption scheme.

**Theorem** **1.** *If Sample-then-Lock is a computational security reusable fuzzy extractor that is $\left(\epsilon_{sec},s_{sec}\right)$-hard with near-liner error, and* Π *is a private-key encryption scheme that achieves indistinguishable encryptions against an eavesdropper, the hybrid encryption scheme $\Pi^{hy}$ of Sample-then-Lock, and any Pi is also a computational security encryption scheme.*

**Proof.**  Before formally proving the above theorem, some intuitive expressions are given. The notation $X\overset{c}{\equiv }Y$ denotes the condition where an adversary cannot distinguish between two distributions *X* and *Y* in polynomial time. Let $W_{j}$ be input to *Gen*. Moreover, let *R* (resp., *P*) denote the uniform string (resp., Helper Data) output by *Gen*. The fact that Sample-then-Lock is computational security means that

$$\left(W_{j},P,R\right)\overset{c}{\equiv }\left(W_{j},P,U\right),$$

where *U* denote the uniform distribution over ${\left\{0,1\right\}}^{\kappa }$. Likewise, suppose the chosen symmetric-key encryption Π provides indistinguishable encryptions against an eavesdropper. In that case, it implies that for any pair of messages, $m_{0}$ and $m_{1}$ generated by an adversary A, the encryptions of $m_{0}$ and $m_{1}$ under a randomly chosen key *k* are computationally indistinguishable, denoted by $Enc_{k}\left(m_{0}\right)\overset{c}{\equiv }Enc_{k}\left(m_{1}\right)$.

Proving the computational security of hybrid encryption scheme $\Pi^{hy}$ means proving that

$$\left(W_{j},P,Enc_{k_{R}}\left(m_{0}\right)\right)\overset{c}{\equiv }\left(W_{j},P,Enc_{k_{U}}\left(m_{1}\right)\right)$$

for $m_{0},m_{1}$ output by a probabilistic polynomial-time (PPT) adversary A. That is to say, for any PPT adversary $A^{hy}$ and $HyK_{A^{hy},\Pi^{hy}}^{eav}\left(n\right)$, the goal of the security proof is to have a negligible function *negl*

$$Pr\left[HyK_{A^{hy},\Pi^{hy}}^{eav}\left(n\right)=1\right]\leq \frac{1}{2}+negl\left(n\right).$$

By definition of the experiment,

$$\begin{array}{cc} Pr[HyK_{A^{hy},\Pi^{hy}}^{eav}\left(n\right)=1] & =\frac{1}{2}\cdot Pr\left[A^{hy}\left(W_{j},P,Enc_{k_{R}}\left(m_{0}\right)\right)=0\right]\\ \; & +\frac{1}{2}\cdot Pr\left[A^{hy}\left(W_{j},P,Enc_{k_{R}}\left(m_{1}\right)\right)=1\right]. \end{array}$$

**Figure 9:**
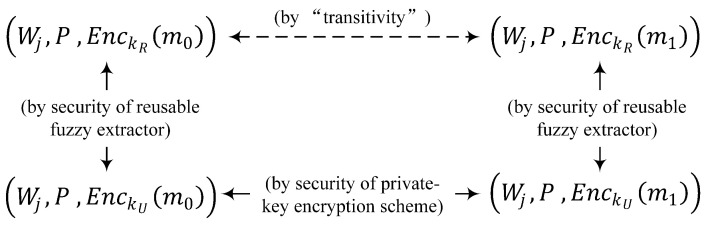
High-level structure of the proof of Theorem 2 (the arrows represent indistinguishability).

The proof proceeds in three steps (As shown in Figure 9).

Step 1. $\left(W_{j},P,Enc_{k_{R}}\left(m_{0}\right)\right)\overset{c}{\equiv }\left(W_{j},P,Enc_{k_{U}}\left(m_{0}\right)\right),$ where on the left $k_{R}$ is obtained from *R* and on the right $k_{U}$ is from *U*, which is a uniform distribution over ${\left\{0,1\right\}}^{\kappa }$. This follows by direct reduction since the computational security of Sample-then-Lock implies that the output value of the fuzzy extractor *R* cannot be distinguished from the uniform distribution *U*, that is, $k_{R}$ cannot be distinguished from $k_{U}$.

Consider the PPT adversary $A_{1}$ attacker of Sample-then-Lock.

Adversary $A_{1}$:

(1) Give $\left(W_{j},P,\hat{k}\right)$ to $A_{1}$.
(2) $A_{1}$ computes $c\leftarrow Enc_{\hat{k_{R}}}\left(m_{0}\right)$, gives <P,c> to $A^{hy}$, the attacker of $\Pi^{hy}$. Then, $A^{hy}$ outputs the bit $b^{{}^{\prime }}$.

In the experiment to attack Π, if b=0, then $A_{1}$ receives $\left(W_{j},P,\hat{k}\right)$ as input, where *P* is generated by the **Gen** algorithm and $\hat{k}$ is derived by applying privacy amplification to *R*. This indicates that $A^{hy}$ receives a ciphertext in the form of $<P,c>=<P,Enc_{k_{R}}\left(m_{0}\right)>$. So,

$$Pr\left[A_{1}\;outputs\;0|b=0\right]=Pr\left[A^{hy}\left(W_{j},P,Enc_{k_{R}}\left(m_{0}\right)\right)=0\right].$$

On the other hand, when b=1 in experiment $RFE_{A_{1},STL}\left(n\right)$ then $A_{1}$ is given $\left(W_{j},P,\hat{k}\right)$ where $\hat{k}$ is obtained by *U* through privacy amplification. Note that *U* is a uniform distribution over ${\left\{0,1\right\}}^{\kappa }$. This indicates that $A^{hy}$ receives a ciphertext in the form of $<P,Enc_{k_{U}}\left(m_{0}\right)>,$ and

$$Pr\left[A_{1}\;outputs\;1|b=1\right]=Pr\left[A^{hy}\left(W_{j},P,Enc_{k_{U}}\left(m_{0}\right)\right)=1\right].$$

As a computational security reusable fuzzy extractor, Sample-then-Lock has a negligible function **negl**${}_{1}$ such that

$$\begin{array}{cc} \frac{1}{2} & +negl_{1}\left(n\right)\geq Pr\left[RFE_{A_{1},STL}\left(n\right)\right]=1\\ \; & =\frac{1}{2}\cdot Pr\left[A_{1}\;outputs\;0|b=0\right]+\frac{1}{2}\cdot Pr\left[A_{1}\;outputs\;1|b=1\right]\\ \; & =\frac{1}{2}\cdot Pr\left[A^{hy}\left(W_{j},P,Enc_{k_{R}}\left(m_{0}\right)\right)=0\right]\\ \; & +\frac{1}{2}\cdot Pr\left[A^{hy}\left(W_{j},P,Enc_{k_{U}}\left(m_{0}\right)\right)=1\right]. \end{array}$$

Step 2. $\left(W_{j},P,Enc_{k_{U}}\left(m_{0}\right)\right)\overset{c}{\equiv }\left(W_{j},P,Enc_{k_{U}}\left(m_{1}\right)\right).$ This equation is derived by considering that $\Pi^{{}^{\prime }}$ achieves indistinguishable encryptions in the presence of an eavesdropper.

**Adversary $A_{2}$:**

(1) $A_{2}$ chooses $W_{j}$ and runs Gen on its own to generate $\left(P,k_{R}\right)$.
(2) $A_{2}$ runs $A^{hy}$ to encrypt $m_{0},m_{1}$. These are produced by $A_{2}$, which also returns a ciphertext *c*.
(3) $A_{2}$ gives <P,c> to $A^{hy}$. Then, $A^{hy}$ outputs the bit $b^{{}^{\prime }}$.

In the experiment $PrivK_{A_{2},\Pi }^{eav}\left(n\right)$, if b=0, the adversary $A_{2}$ is provided with a ciphertext *c* which is an encryption of $m_{0}$ using a key $k_{U}$. So $A^{hy}$ is given $P,Enc_{k_{U}}\left(m_{0}\right)$ and

$$Pr\left[A_{2}\;outputs\;0|b=0\right]=Pr\left[A^{hy}\left(W_{j},P,Enc_{k_{U}}\left(m_{0}\right)\right)=0\right].$$

In contrast, in the $PrivK_{A_{2},\Pi }^{eav}\left(n\right)$ experiment with b=1, the adversary $A_{2}$ is provided with a ciphertext that encrypts the message $m_{1}$ using $k_{U}$. This means $A^{hy}$ is given $<P,Enc_{k_{U}}\left(m_{1}\right)>$ and

$$Pr\left[A_{2}\;outputs\;1|b=1\right]=Pr\left[A^{hy}\left(W_{j},P,Enc_{k_{U}}\left(m_{1}\right)\right)=1\right].$$

Indistinguishable encryption of Π in the presence of an eavesdropper means that a negligible function negl${}_{2}$ exists such that

$$\begin{array}{cc} A\frac{1}{2} & +negl_{2}\left(n\right)\geq Pr[PrivK_{A_{2},\Pi }^{eav}\left(n\right)=1\\ = & \frac{1}{2}\cdot Pr\left[A_{2}\;outputs\;0|b=0\right]+\frac{1}{2}\cdot Pr\left[A_{2}\;outputs\;1|b=1\right]\\ = & \frac{1}{2}\cdot Pr[A^{hy}\left(W_{j},P,Enc_{k_{U}}\left(m_{0}\right)\right)=0\\ \; & +\frac{1}{2}\cdot Pr\left[A^{hy}\left(W_{j},P,Enc_{k_{U}}\left(m_{1}\right)\right)=1\right]. \end{array}$$

Step 3. Exactly as in the case of Step 1, there has

$$\left(W_{j},P,Enc_{k_{R}}\left(m_{1}\right)\right)\overset{c}{\equiv }\left(W_{j},P,Enc_{k_{U}}\left(m_{1}\right)\right)$$

by relying again on the computational security of Sample-then-Lock.

By following the same steps used to prove *Step 2*, there is a negligible function **negl**${}_{3}$ such that

$$\begin{array}{cc} \frac{1}{2} & +negl_{3}\left(n\right)\geq Pr\left[RFE_{A_{3},STL}\left(n\right)=1\right]\\ = & \frac{1}{2}\cdot Pr\left[A_{3}\;outputs\;0|b=0\right]+\frac{1}{2}\cdot Pr\left[A_{3}\;outputs\;1|b=1\right]\\ = & \frac{1}{2}\cdot Pr\left[A^{hy}\left(W_{j},P,Enc_{k_{R}}\left(m_{1}\right)\right)=0\right]+\frac{1}{2}\cdot Pr\left[A^{hy}\left(W_{j},P,Enc_{k_{U}}\left(m_{1}\right)\right)=1\right]. \end{array}$$

There exists a negligible function *negl* such that

$$\begin{array}{c} \frac{3}{2}+negl\left(n\right)\geq \frac{1}{2}\cdot \\ \left(Pr\left[A^{hy}\left(W_{j},P,Enc_{k_{R}}\left(m_{0}\right)\right)=0\right]+Pr\left[A^{hy}\left(W_{j},P,Enc_{k_{U}}\left(m_{0}\right)\right)=1\right]\right.\\ \left.+Pr\left[A^{hy}\left(W_{j},P,Enc_{k_{U}}\left(m_{0}\right)\right)=0\right]+Pr\left[A^{hy}\left(W_{j},P,Enc_{k_{U}}\left(m_{1}\right)\right)=1\right]\right.\\ \left.+Pr\left[A^{hy}\left(W_{j},P,Enc_{k_{R}}\left(m_{1}\right)\right)=1\right]+Pr\left[A^{hy}\left(W_{j},P,Enc_{k_{U}}\left(m_{1}\right)\right)=0\right].\right) \end{array}$$

by summing Steps 1–3 and using the fact that the sum of three negligible functions is negligible.

Note that

$$Pr\left[A^{hy}\left(W_{j},P,Enc_{k_{U}}\left(m_{0}\right)\right)=0\right]+Pr\left[A^{hy}\left(W_{j},P,Enc_{k_{U}}\left(m_{0}\right)\right)=1\right]=1,$$

because the sum of probabilities of complementary events always is 1. Similarly,

$$Pr\left[A^{hy}\left(W_{j},P,Enc_{k_{U}}\left(m_{1}\right)\right)=0\right]+Pr\left[A^{hy}\left(W_{j},P,Enc_{k_{U}}\left(m_{1}\right)\right)=1\right]=1.$$

Therefore,

$$\begin{array}{cc} \frac{1}{2}+negl\left(n\right) & \geq \frac{1}{2}\cdot \left(Pr[A^{hy}\left(W_{j},P,Enc_{k_{R}}\left(m_{0}\right)\right)=0]\right.\\ \; & \left.+Pr[A^{hy}\left(W_{j},P,Enc_{k_{R}}\left(m_{1}\right)\right)=1]\right)\\ \; & =Pr[HyK_{A^{hy},\Pi^{hy}}\left(n\right)=1, \end{array}$$

proving the Theorem 1.  □

### 5.2. Informal Security Analysis

In addition, some natural properties of superlattice PUFs give the proposed scheme some additional security. Next, the following justify how the desirable security features can be guaranteed based on these properties.

- Insider Attack: In the scheme proposed in this paper, the subgroup key is changed when the subgroup members leave, which guarantees forward secrecy. Subsequently, the members of the current subgroup agree on the new key. The leaving members are prevented from obtaining new challenge sequences and helper data because the sender uses the public-key cryptosystem to encrypt them. That is to say, leaving members cannot obtain the new subgroup key. Thus, insider attacks are blocked.
- Dictionary Attack: The output signal of the superlattice device is unpredictable. Even if an adversary obtains the challenge sequence, they cannot use mathematical methods to infer the output signal (key). Thus, attackers cannot obtain the group key through a dictionary attack.
- Replay Attack: The superlattice PUF cannot be cloned once prepared, including the physical entity and its electrical characteristics. Even if the third party obtains the challenge sequence from the public channel, obtaining the output signal (key) is impossible by forging, imitating the device, or fitting its function. Furthermore, old responses are discarded after re-keying, and forward secrecy during re-keying is designed to protect the system from such attacks.
- Man-in-the-middle Attack: The group key is established by legal members locally using the twinning superlattice PUF and reusable fuzzy extractor. The attacker does not hold the multi-twinning superlattice PUF device so that attackers cannot tamper with the public shared messages among members to obtain the group key, rendering the attack ineffective.
- Machine Learning Attack: Machine learning attacks usually collect CRPs as training data and run a learning algorithm to obtain a model close to the actual model [2]. However, the CRPs of superlattice PUFs grow exponentially with the length of the challenge sequence, which has strong PUF properties. This feature is due to the structure of the superlattice PUF, which has 50 quantum wells, and each quantum well has four thin layers of materials. The thin layers of materials have fluctuations in the energy level of single atoms. That is, there will be $3^{4}$ variation samples for each thin layer of material. To sum up, the number of various samples of superlattice PUF structure parameters is ${\left(3^{4}\right)}^{50}\approx 3^{200}\approx 2^{318}$, enough to deal with machine learning modeling attacks.
- **Sybil Attack**: In the proposed scheme, each legal member holds a multi-twinning superlattice PUF on the same wafer, which is physically secure and can be cloned once fabricated, neither mathematically nor physically. During the key agreement process, members use the superlattice PUF and reusable fuzzy extractor to locally generate private keys in response to the sender’s challenge sequence and helper data. Therefore, the attacker can not forge the identity. Furthermore, attackers cannot affect the key agreement process by forging the identity.
- **Key-compromise Impersonation (KCI) Attack**: The member generates their private key using the superlattice PUF locally, which ensures that attackers cannot obtain it. If an attacker obtains the member’s private key illegally, they will only get the challenge sequence and helper data after decryption. However, the actual group key can only be obtained locally through the superlattice PUF which the member hold, and the reusable fuzzy extractor. Therefore, the KCI attack is ineffective.

## 6. Limitations & Future Work

The experimental results in this paper are obtained in the laboratory environment rather than the practical system to verify the feasibility of the key agreement scheme based on multiple twinning superlattice PUFs proposed by this paper. In practical applications, there are still limitations as follows. First, the preparation conditions of matched superlattice PUF are relatively strict. Although there may be multiple devices with similar electrical properties (under the same size and shape) on the same wafer, it is impossible to be duplicated (clone) on another wafer under the designed process conditions. Second, the experimental device in this paper is separate. Integrating these separate devices into a high-speed board is challenging in the practical application system. Lastly, the effectiveness of group key update operations is the significant element limiting the scalable of group communication. When membership changes frequently, a more suitable strategy for re-keying is required.

In the future, efforts will be made on the adaptability of superlattice devices, the design and implementation of high-speed boards, and the optimization and improvement of re-keying strategies to meet the needs of business systems for the proposed scheme. For example, wider voltage bands and better wafer twinning properties will be achieved by improving the material structure of superlattice devices. Moreover, the cost of re-keying in the experiment will be estimated to design a more efficient re-keying strategy.

## 7. Conclusions

This paper proposes a multi-party symmetric key agreement technique based on multiple twinning superlattice PUFs that can be synchronized. The generation and reconstruction of the group key are finished with the help of the reusable fuzzy extractor. The information about the key is not transmitted during the key agreement process, ensuring key distribution security. In addition, subgroup communication is established for members who want to communicate individually through public-key encryption, providing scalable membership changes for subgroups. Extending the point-to-point key agreement technology to multipoint networks by taking the chaos synchronization phenomenon among multiple twinning superlattice PUFs can solve the bottleneck problem in key management and promote the integration of superlattice PUF and cryptographic fields.

## Figures and Tables

**Table 1:** Cost of a join/leave request.

Request	Requesting User	Non-Requesting User	Initiator
join	1	1	n−1
leave	0	1	n−1

**Table 2:** Comparison of computational cost.

References	*n* Device Accessing (ms)		
	Group Key	Member Join	Member Leave
[23]	$(T_{H}+\left(\frac{1}{2}+\frac{1}{2^{2}}+\cdots +\frac{1}{2^{m}}\right)T_{Mix})n$	$(T_{H}+T_{Mix})m$	$(T_{H}+T_{Mix})m$
[25]	$(3T_{E}+T_{D}+5T_{XOR}+T_{PUF}+T_{HMAC})n$	$2T_{H}$	$2T_{H}+\left(T_{Mod}\right)n$
[26]	$(T_{H}+T_{XOR}+T_{PUF})n$	$T_{H}+T_{E}$	$(T_{H}+T_{XOR}+T_{PUF})n$
[27]	$(T_{H}+2T_{PUF}+2T_{E}+2T_{D})n$	/	/
This work	$(T_{PUF}+T_{RFE})n$	$(T_{PUF}+T_{RFE}+2T_{E/D})n$	$(T_{PUF}+T_{RFE}+2T_{E/D})n$

## Data Availability

The data required for simulation are generated through experiments.

## Abbreviations

The following abbreviations are used in this manuscript:

KDC	Key Distribution Center
LKH	Logical Key Hierarchy
IoE	Internet of Everything
PUF	Physical Unclonable Function
WSN	Wireless Sensor Networks
AC	Auxiliary Controller
MC	Main Controller
SDN	Software-Defined Networking
BAN	Body Area Network
DoS	Denial of Service
KCI	Key-compromise Impersonation
